# Out-of-pocket expenditure in patients with isolated traumatic brain injury: a two center cohort study, a preliminary report

**DOI:** 10.1186/s12962-026-00716-1

**Published:** 2026-01-12

**Authors:** Harshit Arora, Sanket Patil, Pranav Bhatia, Monty Khajanchi, Bhakti Sarang, Priyansh Nathani, Rohini Dutta, Shilpa Khanna, Lovenish Bains, Niyara Seit-Yagyayeva, Priti Patil, Anurag Mishra, Riya Sawhney, Monali Mohan, Deepa Veetil Kizhakke, Udit Choubey, Anita Gadgil, Nobhojit Roy, IndSurg Collaborative

**Affiliations:** 1 WHO Collaboration Center for Emergency Critical and Operative Care, Program for Global Surgery and Trauma, The George Institute of Global Health, New Delhi, India; 2 Department of Neurosurgery, Brigham and Women’s Hospital, Harvard Medical School, Boston, MA USA; 3 Department of Gastrointestinal Surgery, Seth G.S. Medical College and K.E.M. Hospital, Mumbai, India; 4 Seth G.S. Medical College and K.E.M. Hospital, Mumbai, India; 5 Department of Surgery, Seth G.S. Medical College and K.E.M. Hospital, Mumbai, India; 6 Department of Surgery, Terna Medical College, Navi Mumbai, India; 7 Yale New Haven Hospital, New Haven, CT USA; 8 Sangwari - People’s Association for Equity and Health, Surguja, India; 9 Department of Surgery, Maulana Azad Medical College, New Delhi, India; 10 Department of Anesthesiology and Pain Management, John H. Stroger Hospital of Cook County, Chicago, IL USA; 11 Program in Global Surgery and Social Change, Department of Global Health and Social Medicine, Harvard Medical School, Boston, MA USA; 12 Department of General Surgery, Indraprastha Apollo Hospital, Delhi, India; 13 Max Institute of GI, MAS, Laparoscopic and Robotic Surgery, Max Super Specialty Hospital, Dwarka, Delhi India; 14 Department of General Surgery, Shyam Shah Medical College, Rewa, India; 15 Department of Global Public Health, Karolinska Institute, Stockholm, Sweden

**Keywords:** Traumatic brain injury, Out-of-pocket expenditure, Low- and middle- income countries, Catastrophic health expenditure, Distress financing, Insurance

## Abstract

**Background:**

Traumatic brain injuries (TBI) account for 30–40% of global injury mortality. In India, high out-of-pocket expenditure (OOPE; 58.7% of total health costs) exacerbates catastrophic health expenditures (CHE). This study estimates OOPE, along with the risk of CHE and distress financing, in patients with isolated TBI.

**Methods:**

We conducted a prospective cohort study across two centers, enrolling patients with isolated TBI over one month. Direct (medical and non-medical) and indirect (wage loss) costs were recorded using medical diaries. 3 months post discharge rehabilitation expenses were recorded.

**Results:**

Eighteen participants (mean age: 43 years, eleven males) were included; four had health insurance, and seven were primary earners. The mean OOPE was INR 88,485 comprising INR 58,319 in direct and INR 30,166 in indirect costs. CHE was observed in fifteen participants and distress financing in eleven participants.

**Conclusion:**

Despite subsidized care, the substantial OOPE for isolated TBI underscores the financial burden, and the imperative for equitable, sustainable healthcare policies.

**Supplementary Information:**

The online version contains supplementary material available at 10.1186/s12962-026-00716-1.

## Background

Injuries contribute to five million deaths annually, contributing to 9% of the global mortality rate. Notably, 90% of the deaths due to injuries occur in low- and middle-income countries (LMICs) [1]. Of note, injuries account for 7.5% of the country’s gross domestic product (GDP), while the national healthcare expenditure constitutes only 1.8% of the annual GDP [2, 3].

Traumatic brain injury (TBI), a hidden epidemic and a growing public health concern, contributes to 30–40% of the global injury mortality across all age groups [4]. TBI can result in neurological disabilities or paralysis, placing severe emotional and financial stress on caregivers, and potentially leading to loss of employment [5].

The global economic impact of TBI is estimated to be approximately USD 400 billion annually [6]. TBI poses significant economic challenges by contributing to high direct medical expenses, including inpatient management, medical and investigation costs, and indirect costs, encompassing income loss due to work absence, travel expenses, prolonged nursing care, and rehabilitation costs [7–9]. The cost is further compounded by the patient’s dependency on the caregiver, leading to expenses and loss of economic productivity for the caregiver.

India reports high out-of-pocket expenditures (OOPE), accounting for 58.7% of the total health expenditures. A disproportionate OOPE between the public and private sector exists, with a five times higher burden observed in the latter [10, 11]. When OOPE exceeds a household’s financial capacity, it may contribute to catastrophic health expenditure (CHE), impacting the spending on daily necessities – food, housing, and education. Previous studies conducted in India have reported a 51% and 22% CHE at threshold of 10% and 30%, respectively in patients managed for injuries [1, 12]. Further, distress financing, a coping strategy to meet healthcare expenditure, involves the injury victims selling assets and borrowing money at higher interest rates. Kastor and colleagues reported 32% of patients suffering from injuries opted for distress financing in India. Moreover, they observed the overall risk of distressed financing was twice as high in patients hospitalized for injury management [13, 14].

The current literature highlights the OOPE for overall injury care in India. However, there exists a dearth of studies reporting injury specific OOPE, including TBI [12–17]. Therefore, this study aims to estimate the OOPE, and the risk of CHE and distress financing in patients diagnosed and managed for isolated TBI.

## Methods

### Study design

This is a prospective observational cohort study with a follow-up period of three months.

### Study setting

Participants were recruited from two tertiary-care urban university medical college hospitals situated in Western region of India, namely Seth G.S. Medical College and KEM Hospital and Terna Medical College. These hospitals are equipped with 24/7 emergency services, round-the-clock operation theatres, blood banks, ICUs, and specialists that are available on call to manage all grades of trauma. The selection of these two hospitals was intentional to analyze OOPE, with one public and one private charitable hospital.

The public hospital serves a lower socio-economic population by offering free - consultations, inpatient stays, and most of the medications, while charging subsidized fees for imaging, surgeries, and non-stocked medications. General surgeons primarily manage injured patients. Subspecialties like vascular surgeons, neurosurgeons etc. are available on call in the hospital campus. The private charitable hospital caters primarily to middle- to low-income populations, providing fee-based services with costs varying according to patients’ economic status. The private charitable hospital caters to middle- and low-income patients. Most patients are covered under state, national or employee health insurance schemes. Additionally, private insurance is accepted wherever applicable. For low-income patients who do not qualify for these schemes, the hospital charges a small amount for IPD services and medicines or utilizes its own funds to ensure they receive necessary treatment. Both hospitals offer subsidized to free care to patients below the poverty line.

Data collection was conducted between December 2022 and September 2023 at different time points, following the approval of the respective Institutional Ethics Committees. The participant recruitment period lasted one month with a 3-month telephonic follow-up.

### Inclusion and exclusion criteria

The study was conducted in accordance with the guidelines outlined by the Declaration of Helsinki. Isolated TBI was defined as a head injury with an Abbreviated Injury Scale (AIS) in the head region of two or higher, with minimal injuries to other body regions (AIS of 1 or lower) [18]. All mechanisms of injury leading to isolated TBI were included (Table 1). We included adult patients aged 18 years or older, of any gender, who had acute isolated TBI. Participants could either arrive directly or be referred to the participating hospitals. Patients who were brain-dead on arrival in addition to isolated TBI were excluded from the study.

Informed consent was obtained from participants or their next-of-kin if the patient was unable to consent due to neurotrauma. Written consent was later obtained from the participants if they were able to do so.

### Data collection and sources

Socio-demographic information like age, gender, education level, household income, earning members, whether the injured person was an earning member, and health insurance coverage was recorded. The injury details like mechanism of injury, Glasgow Coma Score (GCS) at admission, and whether surgical procedures were performed was recorded from the indoor papers of the patient. To calculate Out-of-Pocket Expenditure (OOPE), cost details were collected by asking the patient’s relatives about expenses incurred at three stages: (1) the previous hospitals where the patient was initially admitted immediately post injury, (2) the current hospital (participating study hospitals), and (3) post-hospitalization 3 months after discharge. The costs details from the previous hospital admission were recorded based on the relatives’ recall. Participants were provided with a cost diary to record expenses in real time, reducing recall bias for the current hospitalization and follow-up costs. Medical students, trained by the investigators, followed up with patients every day during hospitalization to document ongoing expenses. Post-discharge follow-up costs were collected telephonically every week for three months and asked to send photos of their expense diaries. If they were hospitalized elsewhere, those expenses were also recorded as post-discharge costs.

Three categories of costs were noted: direct medical costs, direct non-medical costs, and indirect costs. The direct medical and non-medical costs particulars are shown in the supplementary material.

Indirect cost was calculated using the Human Capital Approach (HCA) [19]. For unemployed individuals, the minimum daily wage in Mumbai (INR 470/day) was applied [20]. For deceased individuals, productivity loss was calculated up to the age of retirement (60 years). Distress financing included borrowing money (with or without interest), selling assets, and financial contributions from friends or family (with or without repayment). These methods provided insights into how patients managed healthcare costs. CHE was defined as spending 10% or more of the annual household income on their health-related expenses. For sensitivity analysis, thresholds ranging from 10% to 40% were applied, as different studies use varied thresholds [12, 13].

### Statistical analysis

Categorical variables were described using numbers and percentages. Continuous variables were described using mean and standard deviation, and median with their inter-quartile range (IQR). Costs were presented as the mean (average) cost in Indian Rupees (INR) and US Dollars (USD), along with the median cost, and IQR. The conversion rate used was 1 USD = 83.8 INR. Statistical analyses were conducted using Microsoft Office Excel-2021 [21, 22].

### Ethical consideration

Ethics approval to undertake the study was obtained from the Institutional Ethics Committee of Seth GS Medical College and K.E.M Hospital, Mumbai (Ethics No: EC/OA-68/2022) and Terna Specialty Hospital and Research Center, Navi Mumbai (TMCHRC/SURG/2023/IEC). Administrative approvals to collect data were also obtained from concerned authorities of the respective study hospitals.

## Results

A total of eighteen participants were enrolled over a period of one month at the two participating centers. The mean age of patients was 43 years, and eleven were males. The average annual income of the participants’ families was INR 8,02,667 (USD 9,574.90). Only four participants had health insurance (one private and three government insurance), and seven injured participants were the primary earning members of the family. The median duration of hospital stay for the study participants was 6.5 days (Table 1 and Supplementary Table 1).

**Table 1:** Table describing the baseline demographic and clinical characteristics of the participants of the study. The continuous variables are represented with mean (SD), except length of stay and Glasgow coma scale are represented using median (IQR), and categorical variables as number and proportions

Particulars	Frequency (*n* = 18)
Age (Mean ± SD) (in years)	43 ± 14.65
Number of Male Participants (n, %)	11 (61.1%)
Educational Status (n, %)	
No Formal Education	8 (38.9%)
Primary Education	4 (16.7%)
High School/Graduate/ Postgraduate	6 (11%)
Mechanism of Injury (n, %)	
Fall	7 (38.9%)
Road Traffic Accident	8 (44.4%)
Assault	3 (16.7%)
Glasgow Coma Scale (Median, Range)	14 (6–15)
Primary Earners	7 (38.9%)
Average Annual Household Income, INR (USD)	802,667 (USD 9635.8)
Median Annual Household Income, INR (IQR)	360,000 (240,000–765,000)
Health Insurance	4 (22.2%)
Surgical Intervention	6 (33.3%)
Length of Stay (Median, in days)	7.89 ± 3.74
Mortality	1 (5.6%)

ǂ SD = Standard Deviation; n = Number; USD = United States Dollar; INR = Indian Rupee; Q1 = Quartile 1; Q3 = Quartile 3

Twelve participants reported previous hospitalization before being admitted to the participating centers. The mean direct costs at previous hospitalization were INR 13,233 (USD 157.85), primarily consisting of the direct medical costs of INR 10,312 (USD 123) (Table 2).

**Table 2:** The mean and median direct costs of previous hospitalizations

Cost Categories	Mean INR (USD)	Median INR (IQR)	Range INR
Hospitalization Charges	4427 (52.81)	3000 (2500–5500)	0-16000
Consultation Fees	554 (6.61)	0 (0-750)	0-2600
Imaging Investigation Fee	2118 (25.26)	2000 (900–2750)	0-5000
Laboratory Tests	118 (13.34)	550 (250–1200)	0-4800
Surgery	0 (0)	0 (0)	0–0
Medicines	2122 (25.31)	950 (275–2250)	0-12000
**Total Medical OOPE**	**10,312 (123.01)**	**6500 (4650–12200)**	**45-38400**
Travel	2206 (26.31)	1250 (155–4500)	0-6000
Food	500 (5.96)	0 (0-500)	0-3000
Accommodation	200 (2.39)	0 (0)	0-2000
Nutrient Supplements	0 (0)	0 (0)	0–0
**Total Non-Medical OOPE**	**2922 (34.85)**	**2500 (885–3500)**	**0-10000**
**Total OOPE (Medical + Non-Medical)**	**13,233 (157.85)**	**8620 (6580–14625)**	**1545–48,400**

ǂ INR = Indian Rupee; USD = United States Dollar; OOPE = Out of Pocket Expenses (*n* = 12)

For the current hospitalization, the mean direct cost was INR 21,813 (USD 260.1), with the mean direct medical costs of INR 15,819 (USD 188.6) (Table 3).

**Table 3:** The mean and median (IQR) costs of current hospitalization of patients with TBI. These costs primarily include the direct cost. Some patients did not have the segregated medical and non-medical cost but only their total cost was known. Hence, the total medical and non-medical costs may not add up. Payments made through insurance have been deducted

Cost Categories	Mean INR (USD)	Median INR (IQR)	Range INR
Hospitalization Charges	0 (0)	0 (0)	0
Consultation Fee	0 (0)	0 (0)	0
Radiography Fee	3280 (39.23)	3400 (750–4900)	0-7900
Laboratory Tests	0 (0)	0 (0)	0
Surgery	413 (4.93)	0 (0-500)	0-1600
Medicines	3517 (41.95)	3500 (300–4900)	0-11500
**Total Medical OOPE**	**15,819 (188.69)**	**8450 (3625–10825)**	**0-120000**
Travel	2538 (30.27)	1500 (700–3375)	0-7000
Food	2144 (25.57)	1775 (1025–2750)	0-7000
Accommodation	144 (1.72)	0 (0)	0-2100
Nutrient Supplements	150 (1.79)	0 (0)	0-1500
**Total Non-Medical OOPE**	**5994 (71.50)**	**4275 (3225–7450)**	**750-18000**
**Total OOPE (Medical + Non-Medical)**	**21,813 (260.19)**	**12,450 (9838–17655)**	**1500-138000**

ǂ INR = Indian Rupee; USD = United States Dollar; OOPE = Out of Pocket Expenses

The average total 12-week follow up expenditure for the study participants was INR 23,271.1 (USD 277.2). During this period, the medical, travel, and accommodation costs showed an overall higher OOPE at the end of the first week (mean: INR 3,694; USD = 44.0) compared to the twelfth week (mean: INR 1,061.8; USD = 12.6). None of the participants returned to work until two weeks post-injury. The average time from injury to resuming normal daily functioning was six weeks (Supplementary Table 2). According to the HCA, mean indirect costs for study participants was estimated at INR 30,166 (USD 359.8).

The average total OOPE for an isolated TBI was INR 88,485 (USD 1,0555.90). The average total direct medical expenses and non-medical expenses were INR 58,318 (USD 545.30) and INR 12,623 (USD 150.63). Total Indirect cost accounted for 34% of the total OOPE in isolated TBI patients (Table 4). Health insurance covered on average INR 68,000 in four patients. The mean OOPE for the patients who had insurance was INR 161,613, compared to the ones without insurance, INR 61,919. Three of the four patients who were insured underwent surgery. A total of 6 patients underwent surgical intervention. The OOPE for patients who underwent surgery was INR 1,130,148 (USD 1,553) which was more than twice the participants who did not need surgery (INR 61,036; USD 728.4).

**Table 4:** The distribution of direct, indirect costs and total OOPE in this study and their relative proportions

Out-Of-Pocket Expenses	Average Cost, INR (USD)	Median Cost, INR (IQR)	Proportion (%)
Direct Medical Cost	45,696 (545.30)	35,275 (22612–44325)	51.7
Direct Non-Medical Cost	12,623 (150.63)	10,840 (5550–15025)	14.2
Total Direct Cost	58,319 (695.93)	45,027 (28527–60297)	65.9
Total Indirect Cost	30,166 (359.98)	25,335 (16905–33780)	34.1
Total OOPE	88,485 (1055.91)	66,920 (60006–86062)	100.0

ǂ OOPE = Out of pocket expenditure; INR = Indian Rupee; USD = United States Dollar; IQR = Inter Quartile Range; Q1 = Quartile 1; Q3 = Quartile 3

According to the definition of CHE with a 10% threshold, fifteen participants (83.3%) met this criterion, while if the threshold was raised to 25%, then five participants (33.3%) satisfied the CHE criterion and at 40%, two participants (11.11%) met this criterion. Three out of the four patients with insurance faced CHE at 10% threshold and none of them faced CHE at 25% threshold. Eleven of the eighteen participants (61.1%) adopted a distress financing mechanism; ten participants borrowed money, while one participant’s family sold assets (jewelry) to cover the healthcare costs.

## Discussion

This study provides a comprehensive prospective evaluation of OOPE associated with isolated TBI in two tertiary care centers in India, revealing a significant financial burden on affected households. The mean total OOPE was estimated at INR 88,485 (USD 1,055.90). Notably, direct costs, encompassing both medical and non-medical expenses, accounted for 66% of the OOPE, while indirect costs, reflecting lost productivity and related factors, contributed 34%. CHE at a threshold of 10% as defined by the WHO was observed in 83% of participants, with prevalence diminishing at higher thresholds. Further, a substantial population (61%) of families resorted to distress financing mechanisms, including borrowing or selling assets, to meet the healthcare expenses. These findings highlight the dual financial challenges of direct costs and income loss faced by households managing isolated TBI. Of note, the study participants were managed at a government-subsidized and a private charitable hospital, underscoring the high OOPE associated with the injury even in settings designed to reduce costs. This emphasizes the urgent need for targeted policy interventions to mitigate this burden.

In our study, direct costs were a significant contributor to OOPE, which is consistent with the findings in the previous literature [12, 23]. In previous studies, hospitalization duration, particularly admission to neurosurgical intensive care units (ICU), has been identified as a key driver of total costs, with extended stays resulting in significantly higher burden [24]. Surgical intervention is another important factor known to elevate direct OOPE among patients with TBI. In our cohort, patients who underwent surgical management incurred higher OOPE compared with those treated conservatively, a pattern that aligns with findings reported in earlier studies. Additionally, medications have been widely reported as the most substantial component of direct medical costs [25]. Prinja et al. examined the impact of direct economic costs on overall OOPE of patients suffering from injuries and identified medication and procedural expenses as the primary drivers of direct costs, contributing significantly to the economic burden [12]. They further highlighted that direct costs were higher compared to indirect costs, reflecting the substantial economic impact of procedural and hospitalization expenses on patients. Similarly, Hode et al. reported the average direct cost of treating a TBI patient in Sub-Saharan Africa was around INR 20, 235 (241.4 USD), which is like our direct medical in hospital cost (INR 26,131, 311 USD). This similarity highlights the substantial economic burden of OOPE associated with TBI in LMICs [26].

Indirect costs represent the productivity losses experienced by the patients and their caregivers due to illness [27]. In contrast to the findings of our study, studies conducted in Iran and Netherlands reported a higher proportion of indirect costs contributing to OOPE in TBI compared to the direct costs [5, 9]. Several factors may explain this discrepancy. First, the smaller sample size in our study population may have skewed the overall mean indirect costs, potentially underestimating their contribution. Second, differences in TBI severity across study populations could impact indirect costs; in our study, the predominance of patients with mild TBI, who experienced shorter hospital stays and earlier resumption of daily activities, likely contributed to lower indirect costs, as observed in the findings of Asmamaw et al. [28]. Third, while 38.9% (*n* = 7) of participants in our study were primary earners, the majority were secondary earners, further reducing the overall indirect costs relative to direct costs. These findings emphasize the need to consider patient demographics and disease severity when interpreting the economic burden of TBI. Finally, TBI patients in the Netherlands benefit from comprehensive health insurance and social security schemes, while in Iran, hospitalization costs for TBI patients are covered under national laws. This results in minimal direct costs in both contexts; however, indirect costs, such as productivity losses and the caregiver burden, remain largely uncovered by insurance schemes, contributing substantially to the overall economic burden.

As per WHO, CHE is a situation wherein a household allocates more than 10% of its total annual income to healthcare expenses [8]. In our study, 83.3% of participants met the CHE criteria at the predefined 10% threshold. We selected 10% as the primary threshold because it is the standard WHO-recommended starting point and is simple to apply for smaller datasets. To address variability in CHE definitions across the literature and in response to reviewer concerns, we additionally conducted a sensitivity analysis using thresholds ranging from 10% to 40%, observing that the proportion of households experiencing CHE diminished substantially with higher cut-off values [29]. These results are consistent with findings of Gheysvandi et al., who reported catastrophic costs incurred by 45.4% of TBI patients in Iran [5]. The study further identified subgroups with lower catastrophic costs, notably women, retired individuals, and households of the lower socioeconomic strata. This may be due to these groups typically not being primary earners due to cultural considerations in Iran, resulting in lower indirect costs [5]. While our study observed a higher proportion of participants facing CHE, this may reflect differences in healthcare access, socioeconomic conditions, and cultural factors between the two countries. Future multicentric studies should aim to identify these factors to guide targeted interventions and improve financial protection mechanisms for vulnerable populations.

Distress financing is a well-documented coping mechanism in LMICs like India, where households borrow money at interest or sell assets to meet the financial demands of OOP healthcare expenses [14, 30]. Previous studies have identified factors such as socioeconomic stratification, gender inequities and urban-rural divide as contributors to the practice, with the lower socioeconomic strata being disproportionately affected due to their limited financial resilience [14, 31, 32]. In our study, the high prevalence of distressed financing was evident, with 61.1% of the participants resorting to borrowing or selling assets to cover direct medical costs. However, stratification of patient demographics engaging in distressed financing was not feasible due to the small sample size, highlighting an area for further research in larger, more diverse cohorts.

In our study, only four participants had medical insurance who underwent treatment at the private charitable hospital. Three of the four insured patients incurred CHE at 10% threshold, which may be attributed to only a portion of their in-hospital costs covered by insurance. In contrast, expenses related to previous hospitalization, follow-up care, and indirect costs were paid out-of-pocket, further exacerbating the economic strain on their families. State and National health schemes, such as Ayushman Bharat Pradhan Mantri Jan Arogya Yojana (AB-PMJAY), could play a crucial role in reducing OOPE for TBI patients, particularly for lower-income populations in India [33–35]. Although insurance coverage can reduce direct medical costs and lower the risk of CHE, indirect costs often remain substantial, especially for conditions requiring prolonged hospitalization, surgery, or postoperative rehabilitation. Within our study, despite the presence of insurance, increased OOPE among insured participants may also be attributed to the fact that 75% of the insured patients underwent surgical intervention, a factor known to markedly elevate direct medical expenses. The extent to which insurance coverage mitigates CHE, and by what proportion, remains an area for future research given the small number of insured patients in our study. Although both public and private insurance schemes continued to cover TBI-related hospitalization during the study period, public-sector hospitals still experience delays in activating emergency insurance authorization for urgent neurosurgical procedures, whereas private medical institutions can often initiate provisional approvals more efficiently. Additionally, the potential impact of compounding inflation on healthcare costs, as well as the absence of any documented changes in the applicable insurance schemes during the study period, should be considered when interpreting the overall OOPE burden. Addressing these gaps in coverage is crucial to reducing the financial burden on patients and improving healthcare accessibility.

Previous studies have reported that 70% of uninsured patients from lower socioeconomic backgrounds incur CHE [35, 36]. Internationally, similar trends have been observed: a study in Benin found that uninsured status and structural limitations in the healthcare system were linked to higher treatment costs and delays [26]. Relyea-Chew et al. also found a correlation between lack of insurance and increased medical debt and bankruptcy risk, while Corrigan et al. reported improved functional outcomes for individuals who received state-supported financial assistance for medical bills [37, 38]. Owing to the limited number of patients with insurance we could not see if insurance did mitigate OOPE in our study.

This study demonstrated several strengths. First, its prospective design ensured robust data collection. The use of a cost diary further improved data accuracy and minimized recall bias. Second, the inclusion of two hospitals, a public and a charitable private hospital, enhanced the generalizability of the findings by capturing a diverse patient population. However, the study was limited by the small sample size (*n* = 18), which could potentially reduce the statistical power and limit the generalizability of the results. Further, the study sample primarily consisted of patients with mild TBI (66.7%), which may limit the applicability of the findings to more severe cases. Finally, because the study was conducted in a government-funded tertiary-care hospital and a private charitable institution with subsidized fees, the OOPE reported here may underestimate the financial burden experienced in higher-cost private or corporate hospitals. Given that nearly 60% of inpatient care in India is delivered through fee-for-service private hospitals, OOPE for TBI in those settings is likely to be considerably higher than what we observed [39]. Future research should address these limitations by recruiting multiple centers and different severities of TBI. Moreover, studies should explore cost variations across different hospital settings and examine the impact of public and private insurance coverage on out-of-pocket expenses for managing injuries.

## Conclusion

The mean OOPE for managing isolated TBI over a 3-month follow-up period was estimated above USD 1000, with the average OOPE surpassing 10% of the annual family income, despite participants receiving care at centers offering subsidized healthcare services. Additionally, CHE and distressed financing were highly prevalent in our study, underscoring the disproportionate OOPE relative to the annual household income and assets. These findings further emphasize the urgent need for future prospective studies to explore cost variability across different grades of TBI severity and healthcare settings. Additionally, there is a critical need for comprehensive healthcare policies and programs that assess the financial strain associated with TBI, ensuring equitable and sustainable access to care for vulnerable populations.

## Supplementary Information

Below is the link to the electronic supplementary material.

Supplementary Material 1

## Data Availability

The datasets used and/or analysed during the current study are available from the corresponding author upon reasonable request.
