# Supplementary material for: Out-of-pocket expenditure in patients with isolated traumatic brain injury: a two center cohort study, a preliminary report
Source: Cost Eff Resour Alloc. 2026 Jan 12;24:16. doi: 10.1186/s12962-026-00716-1 (PMC12849665; doi:10.1186/s12962-026-00716-1)
Supplement: Supplementary file 1 — Supplementary Material 1 [file 12962_2026_716_MOESM1_ESM.docx]

**SUPPLEMENTARY TABLE**

**Supplementary Table 1: Date-wise cost diary of the out-of-pocket payments made by the patient a. Direct medical cost; b. Indirect medical costs and Total direct costs.**

| Date | Medical out-of-pocket payments, (Total per visit) (A) | | | | | | |
| --- | --- | --- | --- | --- | --- | --- | --- |
|  | A1  Day charges (for hospitalizations only) | A2  Consultation Fee | A3  Radiograph and Other Imaging | A4  Lab Test | A5  Surgery/Other Procedures Cost | A6  Medicines | ΣA1-6  Medical Payments, Total |

| Date | Non-medical out-of-pocket payments, (Total per visit) (B) | | | | | **Out-of-pocket payments (A+B)**  **(Gross)** | Health insurance reimbursement  (C) | Out-of-pocket payments per stay  (A+B-C)  (Net) |
| --- | --- | --- | --- | --- | --- | --- | --- | --- |
|  | B1  Travel | B2  Food during health care visit or hospital stay | B3  Accommodation | B4  Nutritional supplements or additional food as a part of illness (meat, energy drinks, or fruits as recommended by health care staff) | ΣB1-4  Non-medical out-of-pocket payments (Total) | (ΣA1-6) + (ΣB1-4)  Total out-of-pocket payments |  |  |

| **Follow-Up Week** | **Mean Medicine Expenses (INR)** | **Mean Travel and Accommodation Expenses (INR)** |
| --- | --- | --- |
| **Week 1** | 2472.22 | 1222.22 |
| **Week 2** | 2361.11 | 388.89 |
| **Week 3** | 2361.11 | 283.33 |
| **Week 4** | 1777.78 | 255.55 |
| **Week 5** | 1583.33 | 243.33 |
| **Week 6** | 1283.33 | 242.78 |
| **Week 7** | 1247.22 | 242.78 |
| **Week 8** | 1122.22 | 215.0 |
| **Week 9** | 1125.97 | 214.86 |
| **Week 10** | 947.05 | 157.33 |
| **Week 11** | 712.98 | 134.60 |
| **Week 12** | 927.27 | 134.60 |

**Supplementary table 2. The weekly mean medical and travel & accommodation expenses for a follow-up period of 12 weeks.**

ǂINR = Indian Rupee

**Supplementary Table 3: Table describing the demographic characteristics of the study patients admitted across the two hospitals.**

| **Demography** | **Hospital 1** | **Hospital 2** |
| --- | --- | --- |
| **Age (Mean, SD)** | 40 (15.0) | 46 (14.4) |
| **Sex (Female) (n, %)** | 3 (27.27) | 4 (57.14) |
| **Education (n, %)** |  |  |
| *No Formal Education* | 6 (54.55) | 2 (28.57) |
| *Primary and Middle School/SSC* | 3 (27.27) | 1 (14.29) |
| *Diploma/Graduation/HSC* | 2 (18.90) | 4 (57.15) |
| **Injury to Earning Member (n, %)** | 4 (36.4) | 3 (42.8) |
| **Number of Earning Members (Mean, SD)** | 1.5 (0.9) | 1.1 (0.3) |
| **Number of Dependents (Mean, SD)** | 4.3 (1.3) | 4.4 (1.4) |
| **Health Insurance Available (n, %)** | 0 | 4 (57.1) |
| **Number of Hospitalization Days (Median)** | 6 | 7 |
| **GCS (Median)** | 13 | 14 |
| **Surgery (n, %)** | 3 (27.7) | 3 (42.8) |
| **Mechanism of Injury (n)** |  |  |
| *Fall* | 5 | 2 |
| *RTA* | 5 | 3 |
| *Assault* | 1 | 2 |

ǂ SD = Standard Deviation; n = Number; SSC = Secondary School Certificate; HSC = High School Certificate; GCS = Glasgow Coma Scale

ǂǂ SSC is equivalent to completion of 10^th^ grade whereas HSC is equivalent to the completion of 12^th^ grade.
